# Supplementary figures and images for: Long non-coding RNAs (lncRNAs) NEAT1 and MALAT1 are differentially expressed in severe COVID-19 patients: An integrated single cell analysis
Source: medRxiv. 2021 Jul 31:2021.03.26.21254445. Originally published 2021 Mar 29. Preprint. [Version 2] doi: 10.1101/2021.03.26.21254445 (PMC8020982; doi:10.1101/2021.03.26.21254445)

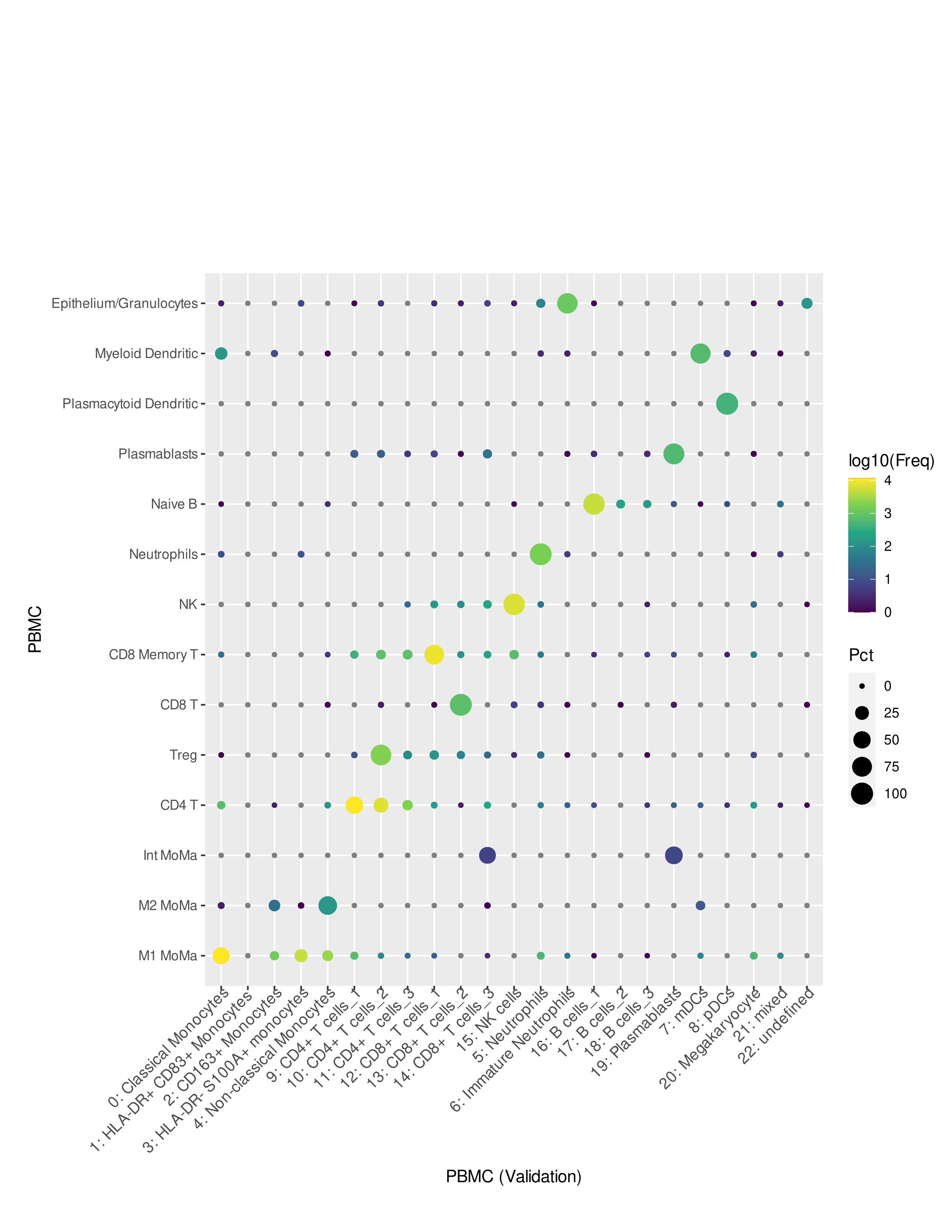

Supplement: Supplement 1 — S1 Figure: Filtering of genes with extremely high residual variance. A. A plot of mean expression levels versus residual variance for all genes detected in dataset. Genes with the highest residual variance are nearly all immunoglobulin and ribosome genes. B. Plot of 100 genes with highest residual variance in descending order. The rate of variance decrease stabilizes after the 21st gene. C. Table of top 21 genes filtered out of downstream analysis due to very high residual variance. S2 Figure: Unbiased clustering of combined BAL and PBMC data reveals common cell types between cohorts and sample conditions. A/B. UMAP plots showing the distribution of all cells in both cohorts within defined cell types. Plot A shows the 15 major cell groups we identified with labels over the cluster centers. Plot B shows the subclusters making up those groups. The major groups are abbreviated as prefixes with a letter suffix indicating subgroup. M1, M2 and Int are the macrophage/monocytes. NK is natural killer cells, E/G is epithelial cells and granulocytes, and E/P/C is epithelia, pneumocyte, and ciliary cells. C. Select markers for major cell groups plotted on the same UMAP projection. This illustrates the specificity of these markers for different regions of the plot corresponding to our respective cell types. D. Dot plot visualization of top markers utilized for identification of each cell cluster. The size of each dot indicates the percentage of cells with detectable expression of each gene, with color indicating expression level. E. Splitting the UMAP by cohort and by severity, with “H” indicating healthy control, “M” indicating mild disease, and “S” indicating severe disease illustrates that cell clusters do not organize according to sample type or patient condition, indicating successful integration of the datasets. S3 Figure: Transferred cell identities correspond to original cell identities from the nasopharyngeal validation set. Dot plot shows cells from the nasopharyngeal [file media-1.zip › FigS4.bmp]

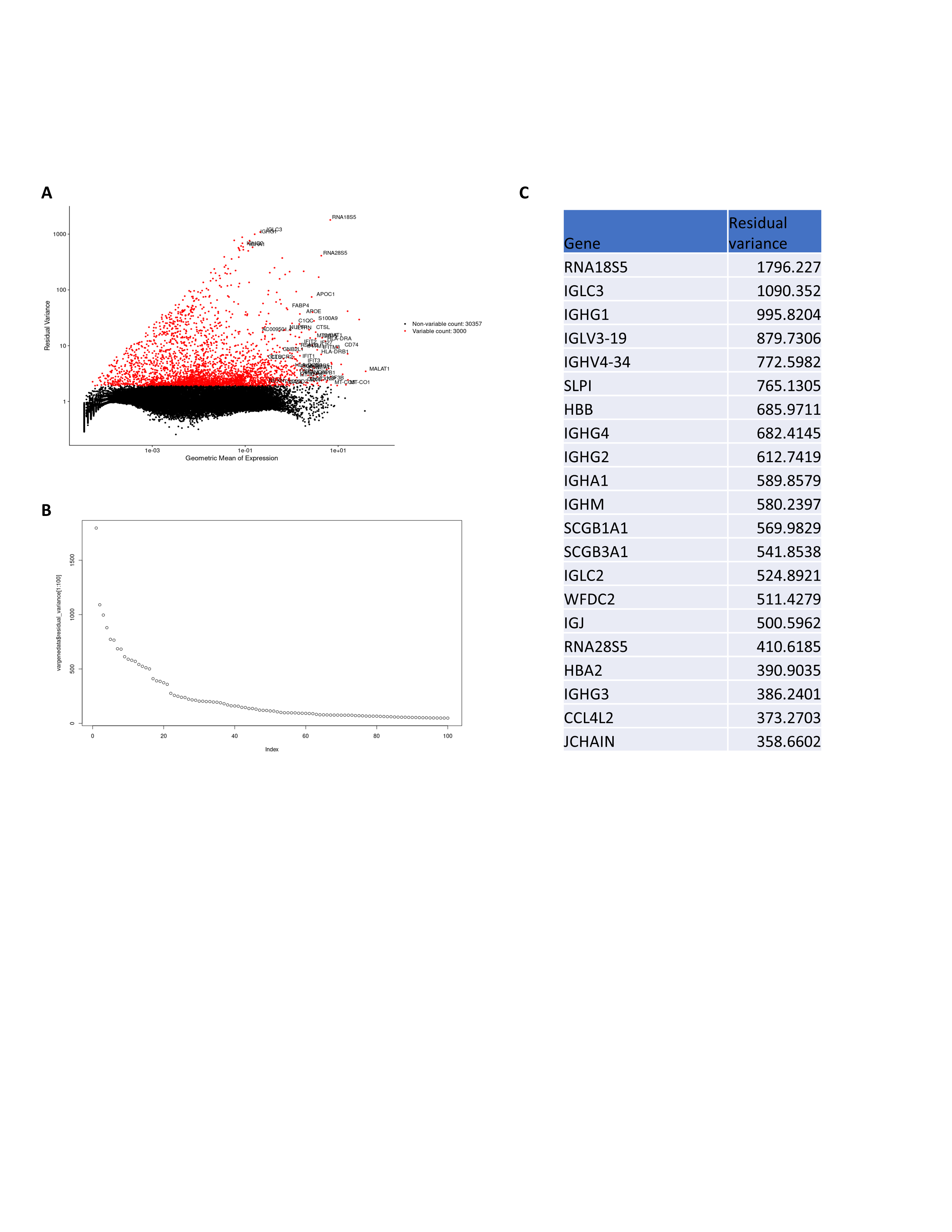

Supplement: Supplement 1 — S1 Figure: Filtering of genes with extremely high residual variance. A. A plot of mean expression levels versus residual variance for all genes detected in dataset. Genes with the highest residual variance are nearly all immunoglobulin and ribosome genes. B. Plot of 100 genes with highest residual variance in descending order. The rate of variance decrease stabilizes after the 21st gene. C. Table of top 21 genes filtered out of downstream analysis due to very high residual variance. S2 Figure: Unbiased clustering of combined BAL and PBMC data reveals common cell types between cohorts and sample conditions. A/B. UMAP plots showing the distribution of all cells in both cohorts within defined cell types. Plot A shows the 15 major cell groups we identified with labels over the cluster centers. Plot B shows the subclusters making up those groups. The major groups are abbreviated as prefixes with a letter suffix indicating subgroup. M1, M2 and Int are the macrophage/monocytes. NK is natural killer cells, E/G is epithelial cells and granulocytes, and E/P/C is epithelia, pneumocyte, and ciliary cells. C. Select markers for major cell groups plotted on the same UMAP projection. This illustrates the specificity of these markers for different regions of the plot corresponding to our respective cell types. D. Dot plot visualization of top markers utilized for identification of each cell cluster. The size of each dot indicates the percentage of cells with detectable expression of each gene, with color indicating expression level. E. Splitting the UMAP by cohort and by severity, with “H” indicating healthy control, “M” indicating mild disease, and “S” indicating severe disease illustrates that cell clusters do not organize according to sample type or patient condition, indicating successful integration of the datasets. S3 Figure: Transferred cell identities correspond to original cell identities from the nasopharyngeal validation set. Dot plot shows cells from the nasopharyngeal [file media-1.zip › FigS1.bmp]

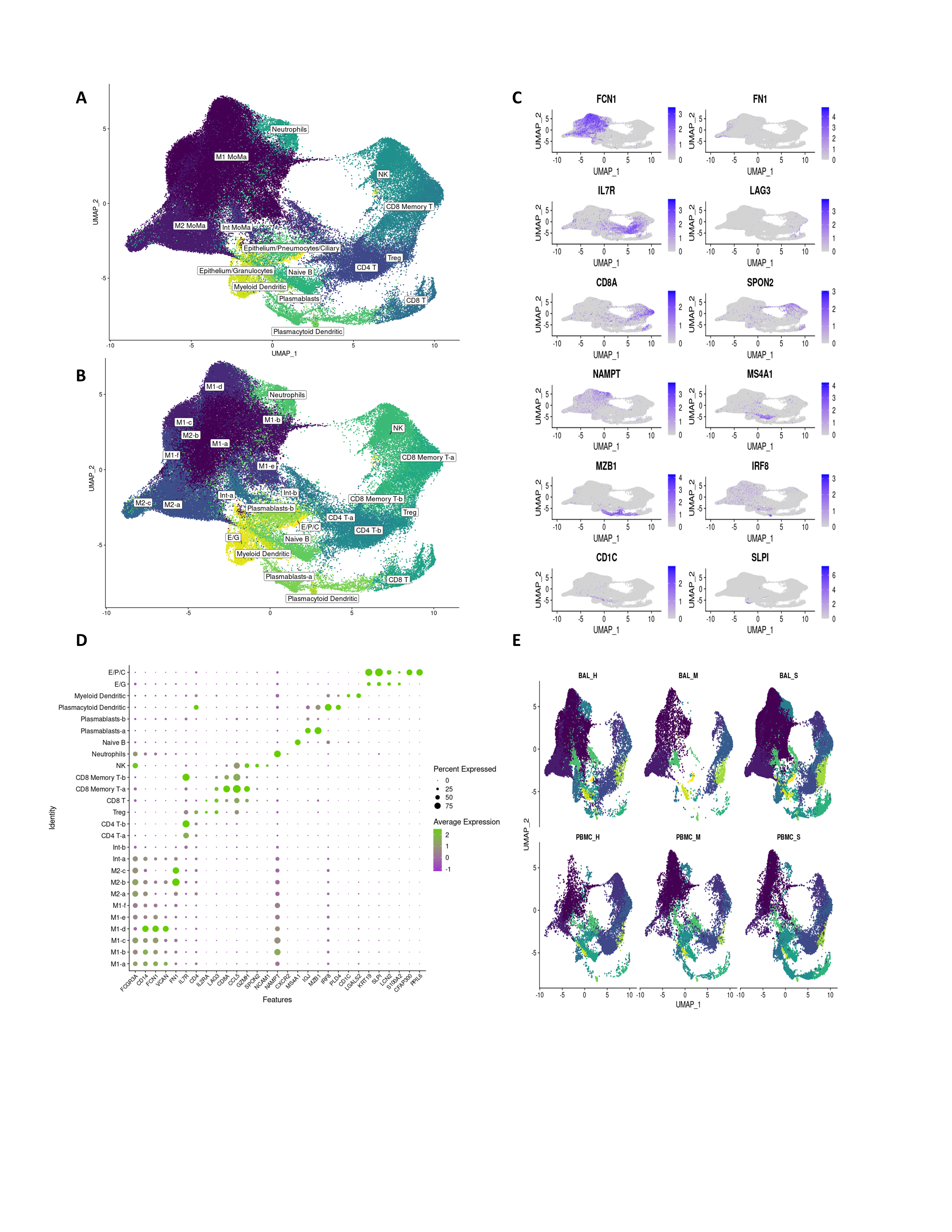

Supplement: Supplement 1 — S1 Figure: Filtering of genes with extremely high residual variance. A. A plot of mean expression levels versus residual variance for all genes detected in dataset. Genes with the highest residual variance are nearly all immunoglobulin and ribosome genes. B. Plot of 100 genes with highest residual variance in descending order. The rate of variance decrease stabilizes after the 21st gene. C. Table of top 21 genes filtered out of downstream analysis due to very high residual variance. S2 Figure: Unbiased clustering of combined BAL and PBMC data reveals common cell types between cohorts and sample conditions. A/B. UMAP plots showing the distribution of all cells in both cohorts within defined cell types. Plot A shows the 15 major cell groups we identified with labels over the cluster centers. Plot B shows the subclusters making up those groups. The major groups are abbreviated as prefixes with a letter suffix indicating subgroup. M1, M2 and Int are the macrophage/monocytes. NK is natural killer cells, E/G is epithelial cells and granulocytes, and E/P/C is epithelia, pneumocyte, and ciliary cells. C. Select markers for major cell groups plotted on the same UMAP projection. This illustrates the specificity of these markers for different regions of the plot corresponding to our respective cell types. D. Dot plot visualization of top markers utilized for identification of each cell cluster. The size of each dot indicates the percentage of cells with detectable expression of each gene, with color indicating expression level. E. Splitting the UMAP by cohort and by severity, with “H” indicating healthy control, “M” indicating mild disease, and “S” indicating severe disease illustrates that cell clusters do not organize according to sample type or patient condition, indicating successful integration of the datasets. S3 Figure: Transferred cell identities correspond to original cell identities from the nasopharyngeal validation set. Dot plot shows cells from the nasopharyngeal [file media-1.zip › FigS2.bmp]

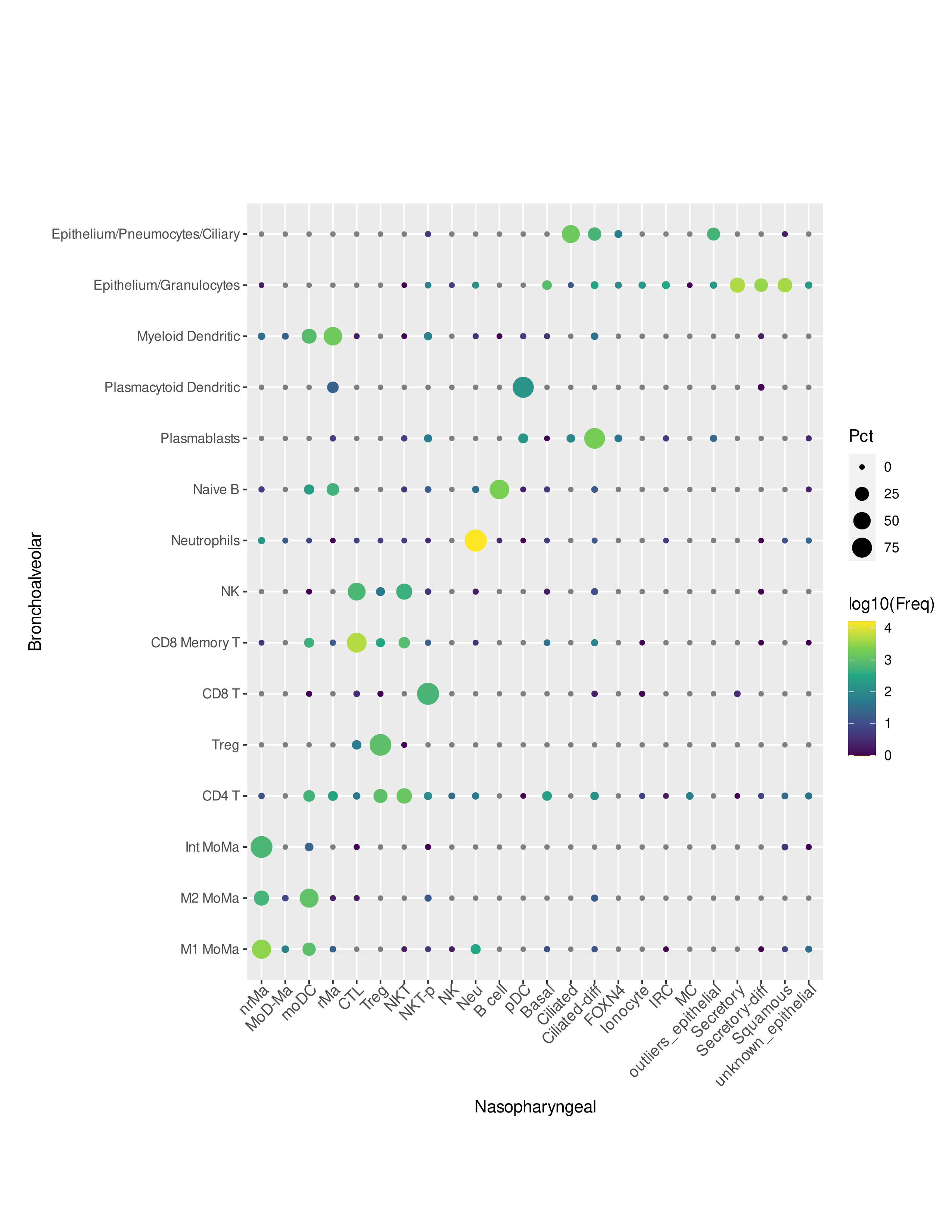

Supplement: Supplement 1 — S1 Figure: Filtering of genes with extremely high residual variance. A. A plot of mean expression levels versus residual variance for all genes detected in dataset. Genes with the highest residual variance are nearly all immunoglobulin and ribosome genes. B. Plot of 100 genes with highest residual variance in descending order. The rate of variance decrease stabilizes after the 21st gene. C. Table of top 21 genes filtered out of downstream analysis due to very high residual variance. S2 Figure: Unbiased clustering of combined BAL and PBMC data reveals common cell types between cohorts and sample conditions. A/B. UMAP plots showing the distribution of all cells in both cohorts within defined cell types. Plot A shows the 15 major cell groups we identified with labels over the cluster centers. Plot B shows the subclusters making up those groups. The major groups are abbreviated as prefixes with a letter suffix indicating subgroup. M1, M2 and Int are the macrophage/monocytes. NK is natural killer cells, E/G is epithelial cells and granulocytes, and E/P/C is epithelia, pneumocyte, and ciliary cells. C. Select markers for major cell groups plotted on the same UMAP projection. This illustrates the specificity of these markers for different regions of the plot corresponding to our respective cell types. D. Dot plot visualization of top markers utilized for identification of each cell cluster. The size of each dot indicates the percentage of cells with detectable expression of each gene, with color indicating expression level. E. Splitting the UMAP by cohort and by severity, with “H” indicating healthy control, “M” indicating mild disease, and “S” indicating severe disease illustrates that cell clusters do not organize according to sample type or patient condition, indicating successful integration of the datasets. S3 Figure: Transferred cell identities correspond to original cell identities from the nasopharyngeal validation set. Dot plot shows cells from the nasopharyngeal [file media-1.zip › FigS3.bmp]
