# Supplementary material for: Long non-coding RNAs (lncRNAs) NEAT1 and MALAT1 are differentially expressed in severe COVID-19 patients: An integrated single cell analysis
Source: medRxiv. 2021 Jul 31:2021.03.26.21254445. Originally published 2021 Mar 29. Preprint. [Version 2] doi: 10.1101/2021.03.26.21254445 (PMC8020982; doi:10.1101/2021.03.26.21254445)
Supplement: Supplement 8 — S8 Table: Comparisons between differentially expressed rDEGs discussed in our results shows strong agreement in validation datasets. A/B. Tables representing the tally of differential expression results of our discussed rDEGs which agreed or disagreed between analysis and validation groups based on the direction of detected differential expression. Tables are split by BAL/nasopharyngeal and PBMC groups. The first three columns correspond to cases where a comparison is not available due to a lack of differential expression detected in the original analysis (na.orig), the validation set (na.val), or both (na.all). The top half of each table reports the results for severe vs mild cases only (SvsM) while the bottom half reports results for all three comparisons: healthy vs mild, healthy vs severe, and severe vs mild. [file media-8.pdf]

A

BAL SvsM	na.orig	na.val	na.all	disagree	agree
M1 MoMa	0	1	0	4	7
M2 MoMa	0	1	0	3	8
NK	0	9	1	1	1
CD4 T	0	4	0	2	6
CD8 Memory T	0	6	0	1	4
**BAL All**	**na.orig**	**na.val**	**na.all**	**disagree**	**agree**
M1 MoMa	0	22	0	4	10
M2 MoMa	0	25	0	3	8
NK	0	33	1	1	1
CD4 T	0	25	0	3	8
CD8 Memory T	0	27	1	1	4

B

PBMC SvsM	na.orig	na.val	na.all	disagree	agree
M1 MoMa	0	1	0	0	5
M2 MoMa	3	0	1	0	1
NK	0	1	0	0	4
CD4 T	0	1	0	1	2
CD8 Memory T	1	0	0	0	2
**PBMC All**	**na.orig**	**na.val**	**na.all**	**disagree**	**agree**
M1 MoMa	0	3	0	4	11
M2 MoMa	3	10	1	0	1
NK	0	3	0	1	11
CD4 T	0	3	1	1	7
CD8 Memory T	2	0	0	0	7
