# Supplementary material for: LncRNAs NEAT1 and MALAT1 Differentiate Inflammation in Severe COVID-19 Patients
Source: medRxiv. 2021 Mar 29:2021.03.26.21254445. Preprint. [Version 1] doi: 10.1101/2021.03.26.21254445 (PMC8020982; doi:10.1101/2021.03.26.21254445)
Supplement: Supplementary file 1 [file media-1.pdf]

A

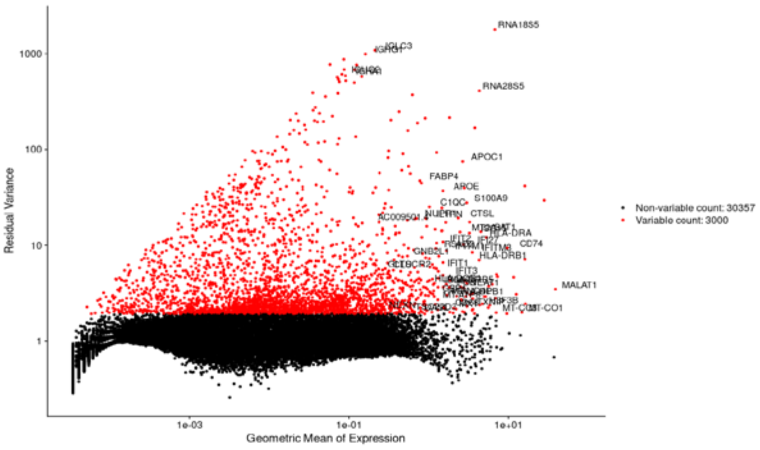

B

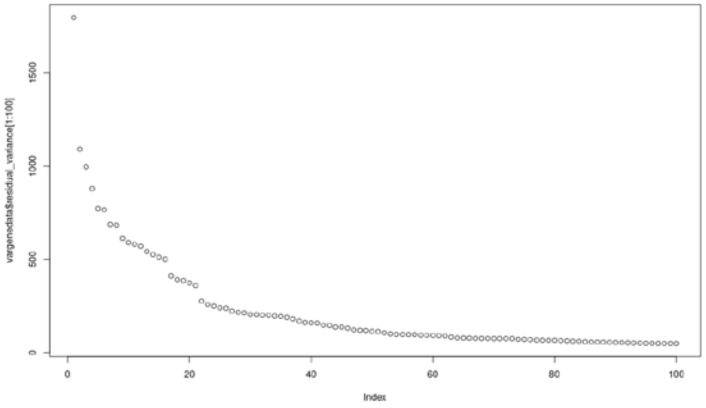

C

Gene	Residual variance
RNA18S5	1796.227
IGLC3	1090.352
IGHG1	995.8204
IGLV3-19	879.7306
IGHV4-34	772.5982
SLPI	765.1305
HBB	685.9711
IGHG4	682.4145
IGHG2	612.7419
IGHA1	589.8579
IGHM	580.2397
SCGB1A1	569.9829
SCGB3A1	541.8538
IGLC2	524.8921
WFDC2	511.4279
IGJ	500.5962
RNA28S5	410.6185
HBA2	390.9035
IGHG3	386.2401
CCL4L2	373.2703
JCHAIN	358.6602

Figure S1: Filtering of genes with extremely high residual variance. A. A plot of mean expression levels versus residual variance for all genes detected in dataset. Genes with the highest residual variance are nearly all immunoglobulin and ribosome genes. **B.** Plot of 100 genes with highest residual variance in descending order. The rate of variance decrease stabilizes after the 21st gene. **C.** Table of top 21 genes filtered out of downstream analysis due to very high residual variance.

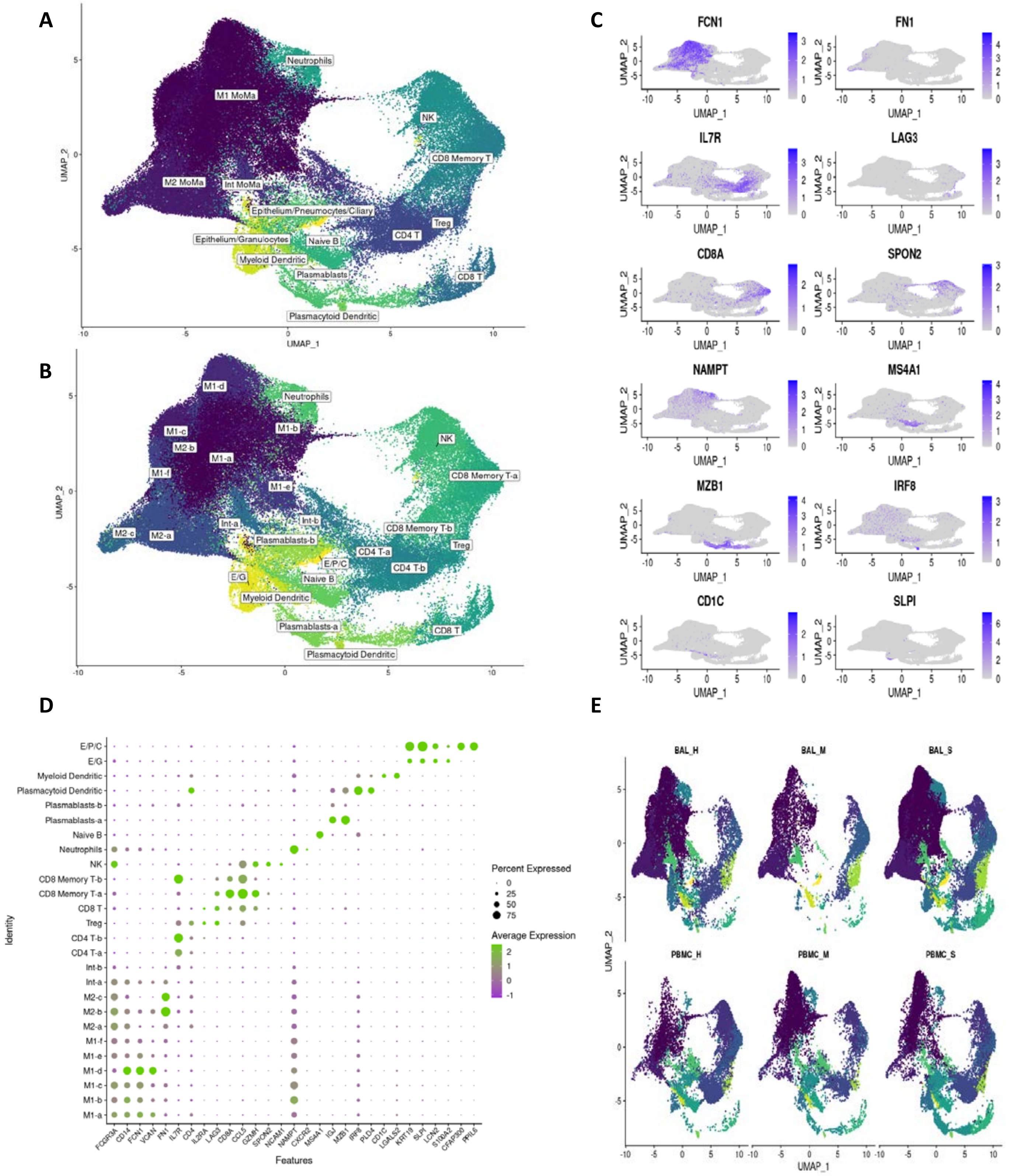

Figure S2: Unbiased clustering of combined BAL and PBMC data reveals common cell types between cohorts and sample conditions. A/B. UMAP plots showing the distribution of all cells in both cohorts within defined cell types. Plot A shows the 15 major cell groups we identified with labels over the cluster centers. Plot B shows the subclusters making up those groups. The major groups are abbreviated as prefixes with a letter suffix indicating subgroup. M1, M2 and Int are the macrophage/monocytes. NK is natural killer cells, E/G is epithelial cells and granulocytes, and E/P/C is epithelia, pneumocyte, and ciliary cells. **C.** Select markers for major cell groups plotted on the same UMAP projection. This illustrates the specificity of these markers for different regions of the plot corresponding to our respective cell types. **D.** Dot plot visualization of top markers utilized for identification of each cell cluster. The size of each dot indicates the percentage of cells with detectable expression of each gene, with color indicating expression level. **E.** Splitting the UMAP by cohort and by severity, with “H” indicating healthy control, “M” indicating mild disease, and “S” indicating severe disease illustrates that cell clusters do not organize according to sample type or patient condition, indicating successful integration of the datasets.

Table S1: Key characteristics of patients within each dataset. Patient information from the BAL and PBMC cohorts used in this analysis. Patients who were intubated or had PaO2/FiO2 ≤ 300 mmHg were classified as severe. In BAL, patient “Mild 3” had only 369 cells recovered after filtering and was only used for initial clustering. Most patients in the BAL cohort. Exact ages were not available in the PBMC cohort. The first patient in this cohort was sampled twice, once while classified as a mild patient, and once after their symptoms worsened and required mechanical ventilation. Several patients in the PBMC cohort received azithromycin, which can have immunomodulatory effects before sample collection.

Subject	Cohort	Age	Gender	Sample Time (Days After Onset)	Interferon	Ribavirin	Methylprednisone	Azithromycin	Outcome
Mild 1	BAL	36	male	11	yes	yes	no	-	Discharged
Mild 2	BAL	37	female	9	no	no	no	-	Discharged
Mild 3	BAL	35	male	13	yes	yes	no	-	Discharged
Severe 1	BAL	62	male	11	yes	yes	no	-	Discharged
Severe 2	BAL	66	male	18	yes	yes	yes	-	Deceased
Severe 3	BAL	63	male	14	yes	yes	yes	-	Deceased
Severe 4	BAL	65	female	15	yes	yes	yes	-	Discharged
Severe 5	BAL	57	female	8	yes	no	yes	-	Discharged
Severe 6	BAL	46	male	11	yes	yes	no	-	Discharged
Mild 1	PBMC	60-69	male	9	-	-	-	yes	Discharged
Severe 1				11					
Mild 2	PBMC	40-49	male	16	-	-	-	no	Discharged
Mild 3	PBMC	50-59	male	15	-	-	-	no	Discharged
Mild 4	PBMC	20-29	male	12	-	-	-	no	Discharged
Severe 2	PBMC	30-39	male	9	-	-	-	yes	Discharged
Severe 3	PBMC	30-39	male	9	-	-	-	yes	Discharged
Severe 4	PBMC	>80	male	2	-	-	-	no	Deceased

Table S2: Demographic characteristics of healthy subjects. All healthy controls used from both the BAL and PBMC cohorts are listed.

Subject	Cohort	Age	Gender
**Healthy control 1**	BAL	38	female
**Healthy control 2**	BAL	24	male
**Healthy control 3**	BAL	22	male
**Healthy control 1**	PBMC	49	female
**Healthy control 2**	PBMC	49	male
**Healthy control 3**	PBMC	36	female
**Healthy control 4**	PBMC	49	male
**Healthy control 5**	PBMC	48	male
**Healthy control 6**	PBMC	37	male
